# Supplementary material for: A novel approach to explore common prime divisor graphs and their degree based topological descriptor
Source: PLoS One. 2025 May 27;20(5):e0323912. doi: 10.1371/journal.pone.0323912 (PMC12111486; doi:10.1371/journal.pone.0323912)
Supplement: Attachment — Submitted filename: Report.pdf [file pone.0323912.s001.pdf]

Report on the article
Title: A novel approach to explore Common Prime Divisor Graphs and their degree based topological descriptor
Manuscript ID: PONE-D-24-59163

The authors have introduced a class of graphs constructed on the divisors of a positive integers, called the common prime divisor graphs. They have explored a few fundamental invariants of these graphs for a specific case. They have also computed various graph-associated topological indices for such graph. In particular, they have presented the degree-based indices for parameters such as alpha, geometric arithmetic, atom-bond connectivity, augmented Zagreb, Albertson, and redefined Zagreb indices.

The way of construction of common prime divisor graph is comparatively a novel approach and has a potential for the future because such graphs can be studied over various other classes of positive integers. The work presented in this article is mathematically correct, the paper is well-written and effectively communicates its outcomes. Some comments about the formatting of the article are given below that needs to be consider before publication.

1. All equations and Theorem are numbered with 0.1, 0.2, etc. Please correct the format.
2. There are a few equations that are too long to be adjusted in one line, please break and adjust accordingly. See proofs of Theorem 0.5 and Theorem 0.6.
3. A general comment: several unnecessary citation are included that can be reduced. See for example [13], [15], [18], [38] and [42].

Recommendation: The article is *recommend* for publication in the PLOS ONE journal.
