# Supplementary material for: A novel approach to explore common prime divisor graphs and their degree based topological descriptor
Source: PLoS One. 2025 May 27;20(5):e0323912. doi: 10.1371/journal.pone.0323912 (PMC12111486; doi:10.1371/journal.pone.0323912)
Supplement: Attachment — Submitted filename: Replies to Reviewers Comment.pdf [file pone.0323912.s002.pdf]

Response to Reviewers

Article ID: PONE-D-24-59163

Article Title: A novel approach to explore Common Prime Divisor Graphs and their degree based topological descriptor

Dear Reviewer
Thank you for your valuable comments and suggestions.

Point Wise Replies to the Reviewers Comments

Comments 1: All equations and Theorem are numbered with 0.1, 0.2, etc. Please correct the format.

Reply: We have corrected the format as required.

Comments 2: There are a few equations that are too long to be adjusted in one line, please break and adjust accordingly. See proofs of Theorem 0.5 and Theorem 0.6.

Reply: We have broken the line and adjusted accordingly.

Comments 3: A general comment: several unnecessary citations are included that can be reduced. See for example [13], [15], [18], [38], and [42].

Reply: We have removed unnecessary citations in the revised version and updated it.

Dr. Moin Akhtar Ansari
(Associate Professor)
Dept. of Mathematics
College of Science
Jazan University, P.O. Box. 114
Jazan 45142
Kingdom of Saudi Arabia
